# Comparison of Properties of Hardfaced Layers Made by a Metal-Core-Covered Tubular Electrode with a Special Chemical Composition

**DOI:** 10.3390/ma13235445

**Published:** 2020-11-29

**Authors:** Artur Czupryński

**Affiliations:** Department of Welding Engineering, Faculty of Mechanical Engineering, Silesian University of Technology, Konarskiego 18A, 44-100 Gliwice, Poland; artur.czuprynski@polsl.pl

**Keywords:** iron-based alloy, hardfacing, surfacing, cladding, tubular hardfacing electrode, FCAW-GS, FCAW-SS, MMAW, wear-resistant steel, wear plate, abrasion, tribology

## Abstract

In this article, the results of research on the metal-mineral-type abrasive wear of a wear-resistant plate made by a tubular electrode with a metallic core and an innovative chemical composition using the manual metal arc hardfacing process were presented. The properties of the new layer were compared to the results of eleven wear plates manufactured by global suppliers, including flux-cored arc welding gas-shielded (FCAW-GS, Deposition Process Reference Number: 138), flux-cored arc welding self-shielded (FCAW-SS, Deposition Process Reference Number: 114), automated hardfacing, and manual metal arc welding (MMAW, Deposition Process Reference Number: 111) hardfacing T Fe15 and T Fe16 alloys, according to EN 14700:2014. Characterization of the hardfaced layers was achieved by using hardness tests, optical microscopy, confocal microscopy, scanning electron microscopy, and EDS (Energy Dispersive Spectroscopy) and X-ray diffraction analyses. Based on wear resistance tests in laboratory conditions, in accordance with ASTM G65-00: Procedure A, and surface layer hardness tests, in accordance with PN-EN ISO 6508-1, the wear plates most suitable for use in metal-mineral conditions were chosen. The results demonstrated the high metal-mineral abrasive wear resistance of the deposit weld metal produced by the new covered tubular electrode. The tubular electrode demonstrated a high linear correlation between the surface wear resistance and the hardness of the metal matrix of the tested abrasive wear plates. In addition to hardness, size, shape, the dispersion of strengthening phases, and the base metal content, depending on hardfacing technology and technological parameters, impact wear resistance is represented by volumetric loss caused by effect-free or constrained dry abrasive medium contact. The presented results can be used in machine part material selection and wear planning for applications in inspection, conservation, and regeneration interval determination. The obtained results will be applied in a real-time wear rate prediction system based on the measurement of the working parameters.

## 1. Introduction

The intensive wear of machine and apparatus parts in mining, quarrying, petrochemical, metallurgical, cement, construction, and power generation industries, among others, drives increasing demand for wear-resistant plates and liners [1,2,3,4,5].

Wear-resistant materials are dedicated alloys with supreme hardness that are simultaneously weldable and resistant to moderate impact loading. Contemporary wear-resistant steels, due to high metallurgical purity, are characterized by high strength, good weldability, workability, and acceptable machinability. The production costs of these steels, however, remain high [6,7,8,9,10,11].

Among the alternatives to the above-mentioned cases are prefabricated wear-resistant composite plates manufactured mainly by automated arc hardfacing, vacuum furnace powder melting, or metallurgical bonding of the base carbon steel plate with a wear-resistant surface layer. Hardfaced wear-resistant plates demonstrate significantly better wear resistance compared to wear-resistant hardened plates [12,13,14,15,16,17,18]. The gains from hardfaced composite wear plates primarily include increased durability, reduced time and cost of repairs, and increased machine operation safety [15,19,20].

The chemical composition and microstructure of the surface layer are chosen to achieve the highest wear resistance and durability of the finished machine and apparatus components. The impact of alloying elements on the hardness of the surface layer is diverse. For wear-resistant steels, high hardness is achieved by a melting process in suitable metallurgical conditions and the addition of set quantities of carbon, manganese, chromium, niobium, molybdenum, and thermal hardening. For wear-resistant composite plates, surface hardness is achieved by the formation of carbon metal compounds in the form of discrete hard particles, known as carbides [6,21,22].

Iron-chromium-carbon alloys are used in conditions where abrasion resistance is required. Their unique abrasive wear resistance results primarily from high volume and the decomposition of the hard carbide fraction in the weld metal matrix. The study of Fe-Cr-C alloy microstructures has demonstrated that these types of materials contain hypoeutectic, eutectic, and hypereutectic structures. Alloys containing 1.8–3.6 mass% carbon and 11–30 mass% chromium are superior in terms of wear, corrosion, and oxidation resistance, and have been adopted as abrasion resistance materials for wear-resistant parts in the mining industry. Primary carbides form in large amounts at higher carbon concentrations. High chromium and carbon contents in weld metal promote the formation of extreme high-hardness chromium carbide particles (1700 HV), which are embedded in significantly more ductile yet hard metal matrices with an average hardness of approximately 700 HV (60 HRC). Due to the presence of these constituents, a hardness level from 600 HV to 840 HV (55–65 HRC) in the surface layer is achieved [6,18].

The wear resistance achieved by hardfaced layers with carbide compounds (mainly M_7_C_3_ and M_3_C) with hardness in the range of 1500–3000 HV is more than a few times higher than widespread wear-resistant materials [1,23,24].

The formation of alloying carbide elements is not the sole criterion in the consideration for the selection of suitable hardfaced composite wear-resistant plates. The shape and distribution of the carbide are also vital to achieving high wear resistance. The highest wear resistance is achieved when elongated cylindrical chromium carbides are situated perpendicularly to the wear-resistant layer surface. Tightly secured by type and the shape of the metal–ceramic interface, uniformly distributed carbides in a hard metal matrix act as a wear barrier, provided that they are resistant to cracking and pulling from the matrix. The lowest wear resistance is observed when chromium carbides are situated parallel to the layer surface due to the increased chance of carbide cracking and pulling from the metal matrix. The shape and orientation of carbides can be controlled to an extent by cored-wire composition selection and proper hardfacing parameters [25].

The microstructure and hardness of the most important properties of wear-resistant materials contribute to resistance to different types of tribological wear. The hardness of the material is directly dependent on the microstructure and is the easiest material property to measure. However, it is often falsely assumed as the single most important criterion in the assessment of wear-resistant materials. The hardness of the two materials can be identical, whereas their wear resistance can be significantly different due to differences in the microstructure and their impact on the hardness [26]. In the majority of cases, however, the increase in hardness coincides with the increase in wear resistance, especially in hardfaced composite wear plates.

Although there is a wide range of hardfacing electrodes, wires, and filler metals commercially available for protection against abrasion wear, the industry is constantly looking for new material solutions. Chromium-rich hardfacing alloys are commonly used due to their low cost and availability; however, more expensive tungsten- or vanadium-rich alloys offer improved welding properties due to their suitable combination of hardness and toughness. According to Kim et al. [27], covered tubular electrodes with a metallic and carbides core can also be used, especially when abrasion occurs with other wear mechanisms, such as erosion, corrosion, and moderate shock load. The basic surfacing techniques are oxyacetylene powder surfacing using a modular spray-fuse system, manual metal arc surfacing, and submerged arc surfacing. Other surfacing processes can also be used, ranging from conventional techniques, such as gas flame surfacing, to new and modern processes, such as plasma powder transferred arc surfacing and laser metal deposition techniques. The manual metal arc surfacing technique is frequently used for hardfacing applications due to its adaptability and cost-effectiveness.

The maintenance of wear plates, which includes wear assessment and replacement, is a major component of very high operating costs. Several research centers, mainly from the USA, Australia, and China, are currently conducting research on a wear detection system for wear plates in an operational environment, which would improve efficiency, safety, and profitability in the mining sector. Designing such a system, however, requires obtaining significant quantitative and qualitative data on the phenomenon of abrasive wear, which is to be supported by the presented research.

## 2. Experimental Procedures

### 2.1. Aim of the Study

The main purpose of this research was to compare the abrasion resistance of the metal-mineral abrasion of the manual metal arc hardfacing layer of a new self-developed covered tubular electrode with wear-resistant plates from leading manufacturers.

This research obtained selected quantitative and qualitative data to develop an innovative system, enabling the assessment of the wear state of hardfaced wear-resistant plates in industrial conditions through the real-time recording of measurement data, such as the operating temperature and vibration. Real-time wear data processed by a computer system were used to predict maintenance requirements, which would enable industrial operators to properly manage, plan, and reduce the cost of maintenance downtime.

Wear resistance determination was performed according to ASTM G65-00: Procedure A [28]. Hardness measurements were made in accordance with ISO 6508-1:2016 [29]. The wear resistance of selected surface layers was compared to Swedish abrasion-resistant steel with a nominal hardness of 400 HBW (~423 HV). The determination of wear character was made by means of macroscopic, confocal microscopy, and scanning electron microscopy examinations, and the properties of the microstructure were examined by light microscopy and X-ray diffraction.

All of the examined wear-resistant hardfaced composite plates were characterized by:

- Comparable geometric properties, i.e., shape, surface waviness, and surface roughness;
- Lack of unacceptable welding defects;
- Hardfaced layer thickness ≥3 mm;
- Base plate thickness ≥5 mm;
- Suitably low base material content in the hardfaced layer.

The range of performed examinations consisted of:

- Wear test (Procedure A);
- Mass loss during wear test determination;
- Reference sample and the examined surface layers density determination;
- Calculation of relative wear resistance;
- Hardness test of the working surface of plates;
- Comparison of the microstructure of the layers;
- Determination of the linear correlation coefficient between the mean layer hardness and wear resistance of the examined composite plates’ surface layers.

### 2.2. Materials’ Characterization

Examinations were performed on samples from 12 wear-resistant hardfaced composite plates (Table 1) produced by different arc hardfacing processes. The selected surfacing technologies differed in the metal deposition rate, penetration depth, surfacing speed, surfacing overlay quality, and the final cost of the product. For a given product type and quality requirements, quality assurance is mainly determined by the coating application process, the technique and technological conditions of coating application, and the type of additional material.

The advantages of the manual metal arc deposition process are its versatility and the availability of a wide range of hardfacing alloys. The set-up time is short, making the process ideal for small jobs and short production runs. For a working surface, the manual metal arc deposition process has lower deposition rates than the flux-cored arc welding (FCAW) deposition processes, which use a higher current. The process has a low operator duty cycle, with the operator spending significant time changing electrodes and chipping slag. These two factors combine to limit the application of this process, especially if high production rates are required.

The flux-cored arc welding gas-shielded (FCAW-GS) deposition process has the advantage of deeper penetration and a higher charging rate than the manual metal arc process. Thus, the welding process is becoming more economical for jobs in welding workshops. Flux-cored arc welding self-shielded (FCAW-SS) filler metal eliminates the need for external shielding gas and tolerates stronger wind conditions without causing porosity.

The compositions of nine layers contain alloys from group T Fe15, and three layers contain alloys from group T Fe16 (including a hardfaced plate with the name PHWP). The obtained results were compared to the reference abrasion-wear-resistant steel plate (Table 2).

**Figure 1:**
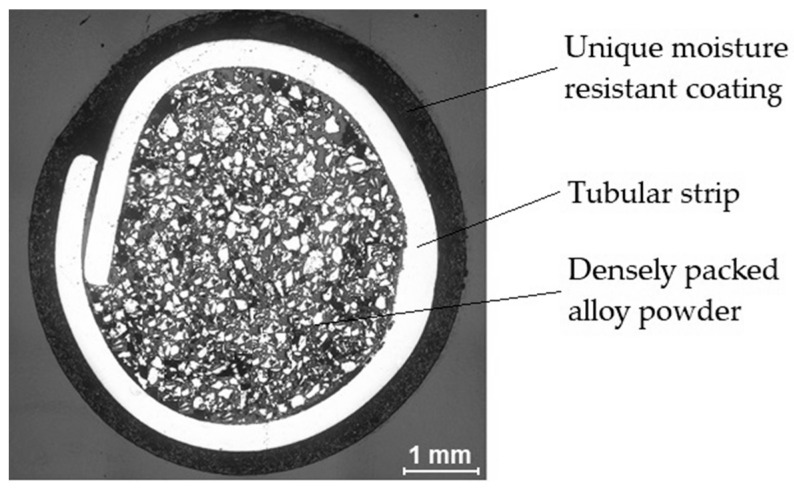
Macrostructure view of the cross-section of the new patented tubular electrode [31].

The wear-resistant plate with the name PHWP was obtained by the arc welding process with a tubular electrode 4 mm in diameter. The metallic core of the electrode has a patented metallic composition with a rutile cover. A cross-sectional view of the electrode is presented in Figure 1.

The samples were cut from the inner regions of the examined hardfaced composite wear-resistant plates and reference material.

### 2.3. Methodology of Research

For each examined sample, a visual test (VT) on the sample surface was performed to identify potential unacceptable welding defects, such as cracks directed longitudinally to the hardfaced seam, other discontinuities, porosity, irregularity of shape, and lack of fusion. Metal-mineral wear resistance was determined by strictly adhering to the procedure disclosed in the ASTM G65 standard [28]. The determination of surface layer structure and other properties was based on metallographic macro- and microscopic examinations, measuring the base material content in the hardfaced layer and the hardness tests on the working surface.

#### 2.3.1. Nondestructive Testing: Visual Testing

Visual testing of the wear plates was performed in accordance with the procedures, materials, and equipment from the ISO 17637 standard [33]. Visual testing was based on a direct inspection of the antiabrasive wear of the hardfaced layer procedure. Before testing, the surface subjected to observation was cleaned.

#### 2.3.2. Hardness Measurements

**Figure 2:**
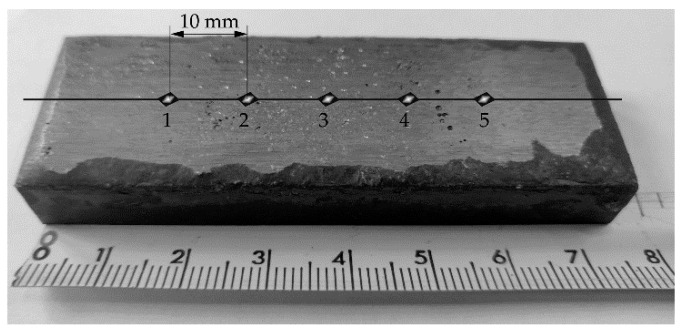
Measurement points location on the wear-resistant layer surface.

The hardness testing of the reference material (Hardox 400 steel) and the hardfaced layer was performed using the Rockwell method Scale C (HRC) on the Nexus 610 RS stationary hardness tester (Innovatest Europe BV, Maastricht, Netherlands). Hardness testing was performed according to ISO 6508 [29]. The test load used was 150 kgf (total test force of 1.471 kN). Hardness tests were performed in five test points on the surface, finished by grinding selected layers manufactured by the arc hardfacing process. The way the measurement points were located on the wear-resistant layer surface is presented in Figure 2.

The carbide’s microhardness measurement was made with the Vickers method at the polished cross-section of the samples. A Sunpoc SMV-1000X (Guizhou Sunpoc Tech Industry Co., Ltd., Guizhou, China) microhardness tester with a diamond pyramidal indenter was used. The load could be varied from 5 to 500 g in fixed steps. The duration was kept at 25 s throughout the study. The examinations were performed in conformity to the ISO 6507-1:2018 standard [34].

#### 2.3.3. Abrasive Wear Test

**Figure 3:**
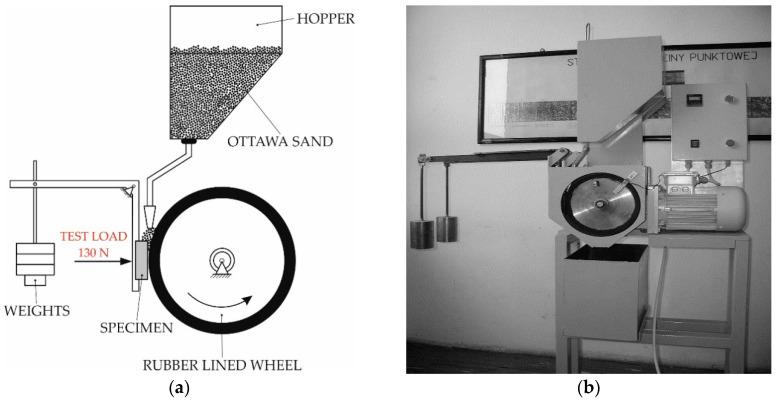
Schematic diagram of ASTM G65, Procedure A: abrasive wear resistance test (**a**) and apparatus overview (**b**) [35].

The metal-mineral wear resistance of all test samples and the reference material (Hardox 400 steel) was carried in accordance with ASTM G 65-00: Standard Test Method for Measuring Abrasion Using the Dry Sand/Rubber Wheel Apparatus, Procedure A (Figure 3) [28].

The rubber wheel test outlined in ASTM G65, first introduced in 1980, is the most widely applied method in determining material wear resistance. The abrasive used in the test procedure is quartz sand with a grain size of 50–70 mesh (0.300–0.212 mm). The abrasive is fed from a gravity hopper with a 250–350 g/min feed rate. The counterface in the test is rubberized by a hard wheel compound with a total dimension of ϕ 228 mm × 12.7 mm. The specimen under investigation is pressed to the counterface by forces of, depending on the procedure chosen, 45 N or 135 N. The test length is determined by the number of wheel revolutions of the counterface and is contained in the range of 100–6000 revolutions (wheel speed of 200 r/min). The measured results comprised the volume of wear and an examination of the worn surface. According to Hejwowski, under the test condition, the worn material is transferred to the surface of the abrasive grains [36]. In the rubber wheel test, the abrasive grains may rotate in the friction zone or temporarily attach to the rubber material. The movement and interaction of the abrasive material are dependent not only on the test parameters (abrasive flux and type, test force, and counterface revolution speed) but also on the hardness of the sample under investigation. The rolling of abrasive grains is facilitated by a low test force and the limited tested sample hardness. For high test forces and an elevated sample hardness, the sliding movement of the abrasive grains is promoted.

**Figure 4:**
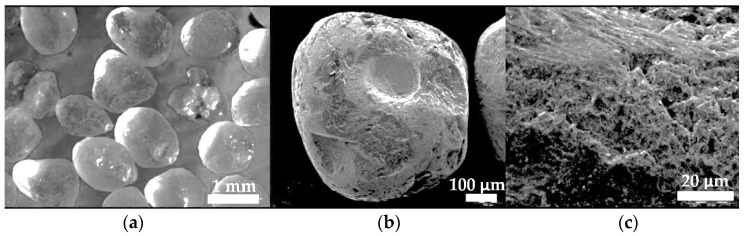
View of the abrasive material particles (A.F.S Testing Sand 50–70) used in ASTM G65-00, Procedure A: abrasive wear resistance test: (**a**) a quartz sand grain fraction, (**b**) a single grain of quartz sand, (**c**) the surface structure of quartz sand grain.

In order to determine the wear resistance of the selected wear-resistant composite plates, two samples (75 mm × 25 mm × 10 mm) were cut from the inner area of the plate. During the test procedure, the counterface wheel made 6000 revolutions, samples were pressed with the force of 130 N, and the abrasive materials’ (A. F. S. Testing Sand 50–70 mesh) expense was 335 g/min (Figure 4). The ASTM G65-00 wear test time was 30 min.

Before and after the test procedure, the samples subjected to wear testing were weighed on a laboratory scale with an accuracy of 0.0001 g. The mean density of the tested hardfaced layers and reference Hardox steel was determined by measuring the weight of three specimens in air and during submersion in liquid at room temperature (Table 3). The measured mass loss during the test procedure, in conjunction with the determined mean density, was used to calculate volumetric loss according to Formula (1). A similar fraction and distribution of the matrix reinforcing phase over the entire cross-section of the surface layer worn during the 30 min test period were assumed.

(1) $$\mathrm{Volume}\;\mathrm{loss}\;\left[{\mathrm{mm}}^{3}\right]=\frac{\mathrm{mass}\;\mathrm{loss}\;\left[g\right]\;}{\mathrm{density}\;\left[\frac{g}{{\mathrm{cm}}^{3}}\right]\;}\times 1000$$

**Figure 5:**
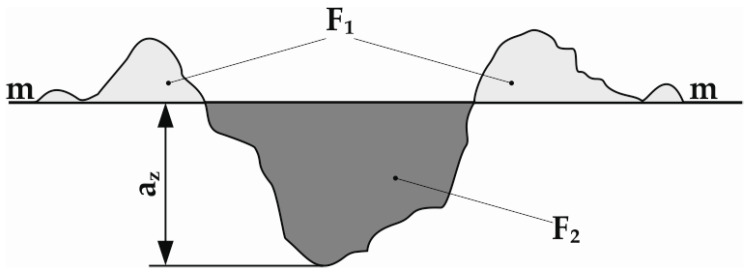
The abrasive wear mechanism criterion: a_z_—groove depth; m-m—reference line.

The abrasive wear mechanism was assessed in accordance with the criterion, which was the quotient of the cross-sectional area of the sum of the two-sided upsets of the material next to the deepest crack F_1_ and crack cavity F_2_ [37,38,39]. The loss of material in the surface layer during abrasive wear was classified as:

- Grooving related to plastic deformation of the contact areas and upset of the material on both sides of the furrow (F_1_/F_2_ = 1);
- Microcutting (F_1_/F_2_ = 0);
- Scratching when the material was partially plastically deformed and partially cut, (0 ≤ F1/F2 ≤ 1), Figure 5.

#### 2.3.4. Metallographic Examination and X-ray Diffraction Analysis

Microscopic examinations were performed on standard metallographic specimens. The etchant chemical composition and etching parameters were determined individually for each hardfaced layer. The observation and acquisition of the macro- and microstructure of the specimens were performed using the Olympus SZX7 (Olympus Corporation, Tokyo, Japan) stereoscopic microscope, the Olympus GX 71 inverse metallographic microscope (Olympus Corporation, Tokyo, Japan), the Zeiss Smartproof 5 confocal microscope (Carl Zeiss AG, Oberkochen, Germany), and the Zeiss Supra 25 scanning microscope (Carl Zeiss AG, Oberkochen, Germany). Precise determination (surface and volumetric) of the surfaced layer chemical composition was performed by means of energy-dispersive spectroscopy (EDS).

**Figure 6:**
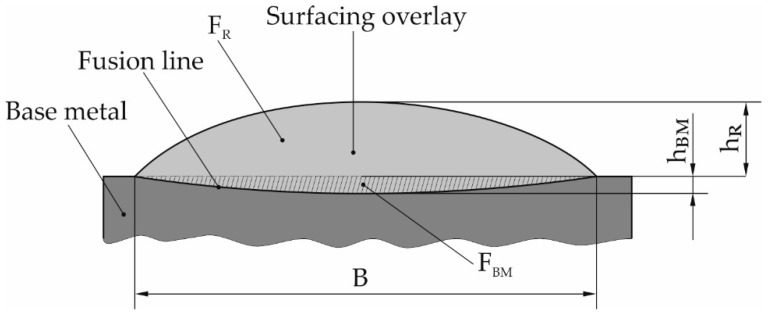
Geometrical parameters of the stringer and weave bead deposits: B—width of the bead face; h_R_—height of the bead reinforcement; h_BM_—base metal penetration depth; F_R_—area of reinforcement; F_BM_—area of base metal melted.

The obtained macroscopic images enabled the determination of surface layer thickness as well as the base material content in the hardfaced layer. The dilution of weld metal, U, was calculated according to Formula (2), as the ratio between F_BM_, the area of the fusion-based metal and a sum of F_BM_ and F_R_, the area of reinforcement, Figure 6.

(2) $$U=\frac{F_{\mathrm{BM}}\;}{F_{R}+F_{\mathrm{BM}}}\times 100\%$$

The X-ray diffraction phase examinations were carried on X’Pert Pro PANalytical (Malvern Panalytical Ltd., Malvern, UK) diffractometer with Cu lamp (λ = 1.54056 nm). The samples were examined in a Bragg–Brentano geometry.

## 3. Results and Discussion

### 3.1. Nondestructive Testing: Visual Testing Results

**Figure 7:**
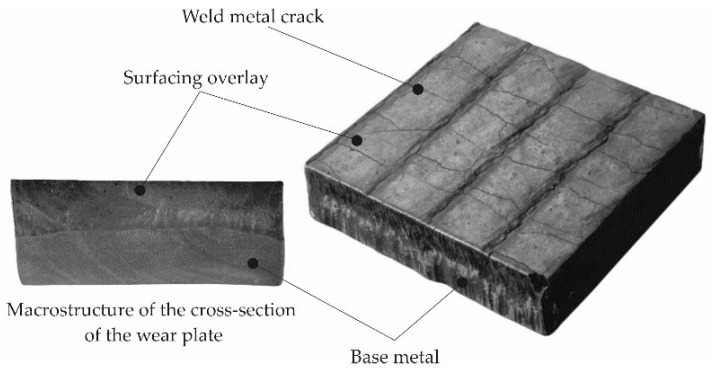
Overview and cross-section of the hardfaced composite wear-resistant plate produced by the automated flux-cored arc welding self-shielded (FCAW-SS) alloy.

During the visual tests of selected hardfaced composite wear-resistant plates produced by the automated FCAW and MMA methods, imperfections of cracks (100) perpendicular to the hardfacing direction and surface spatter (602) were found, as presented in Figure 7.

The hardfacing process is an important process associated with welding; therefore, it is necessary for the verification of the applied hardfacing technology according to accepted standards. For hardfaced composite wear-resistant plates, the ISO 15614-7 [40] standard is suitable. However, applying this standard precisely can be difficult. In acceptance tests, wear-resistant hardfaced layers are often unable to conform to acceptable quality levels and cannot be accepted without remarks. Regular transverse cracks in hardfaced layers contribute to the reduction of stress levels in hardfaced elements and can act as a lubricant reservoir. In the context of product application, transverse cracks in the hardfaced layer can be deemed acceptable.

### 3.2. Hardness Measurements Test Results

**Figure 8:**
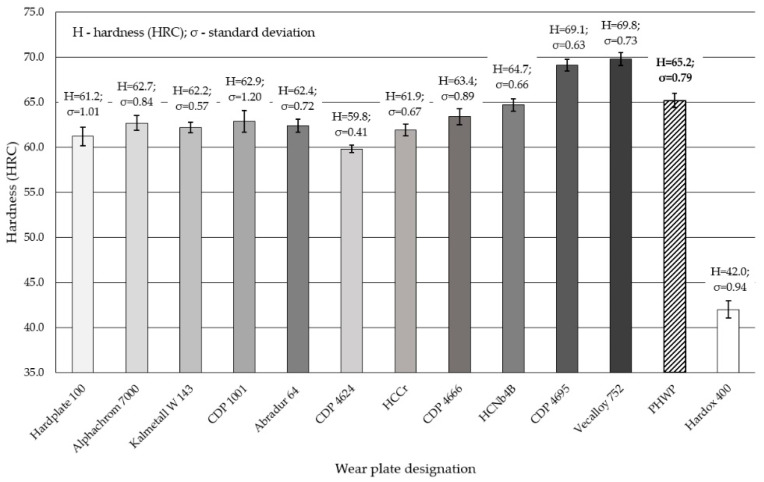
Comparison of the working surface hardness arc hardfaced with alloys from groups T Fe15 and T Fe16: wear-resistant plates and the Hardox 400 reference material.

The results of the mean Rockwell hardness test on the working surface of wear-resistant plates, arc hardfaced with alloys from groups T Fe15 and T Fe16 and subjected to examinations, were significantly higher compared to the Hardox 400 reference material. The results from the five measurement points for each specimen are presented in Figure 8. The hardness measurements placed the hardness of examined plates in the range of 60–70 HRC (from approximately 700 HV to over 900 HV), which is mostly consistent with additional material characteristics and published data in scientific articles [1,41,42,43,44]. Only the hardness of the Alphachrom 700 wear-resistant plate’s working surface was lower than that declared by the manufacturer, which could be caused by high dilution of the weld metal. The average value of the hardness test for the PHWP wear-resistant plate was the lowest among the T Fe16 alloys. The reason for this phenomenon is the high plasticity of the metallic matrix. No major dispersion in the hardness results was observed.

### 3.3. Abrasive Wear Test Results

**Figure 9:**
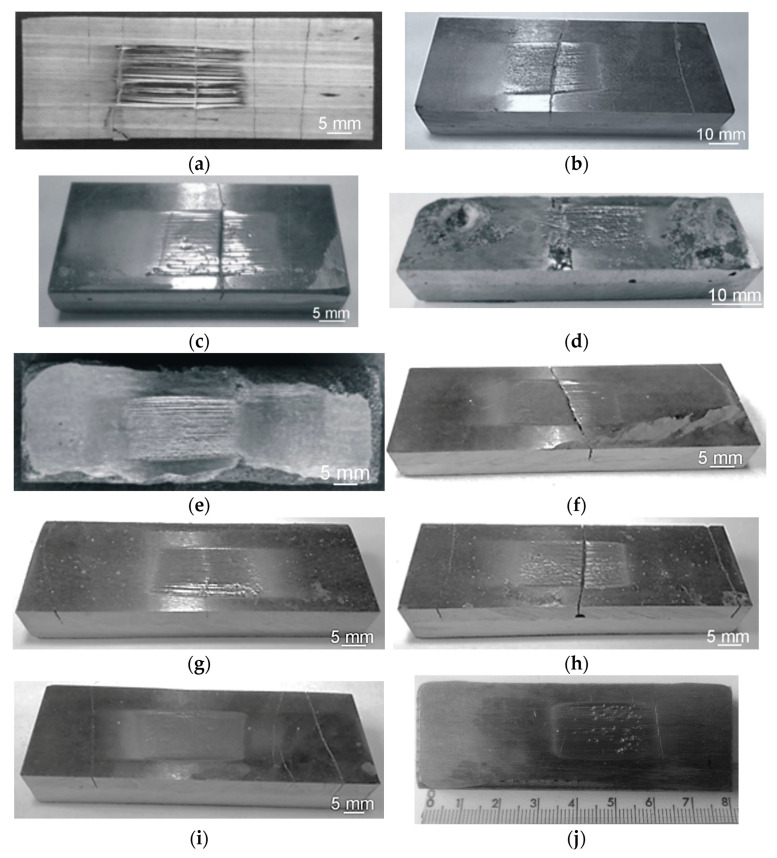

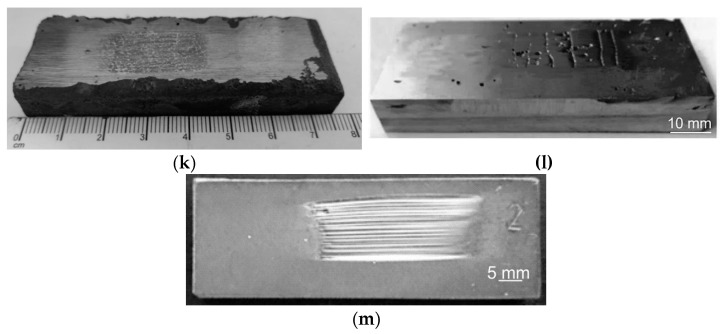
Surface view of the representative sample after the metal-mineral abrasive wear resistance results performed according to ASTM G65-00: Procedure A. Samples from the: (**a**) Hardplate™ 100S wear plate; (**b**) Alphachrom 7000 wear plate; (**c**) Kalmetall W 143 wear plate; (**d**) CDP^®^ 1001 wear plate; (**e**) Abradur 64 wear plate; (**f**) CDP^®^ 4624 wear plate; (**g**) HCCr wear plate; (**h**) CDP^®^ 4666 wear plate; (**i**) HCNb4B wear plate; (**j**) CDP^®^ 4695 wear plate; (**k**) Vecalloy 752 Plate wear plate; (**l**) PHWP wear plate; (**m**) Hardox 400 abrasion-resistant steel.

**Figure 10:**
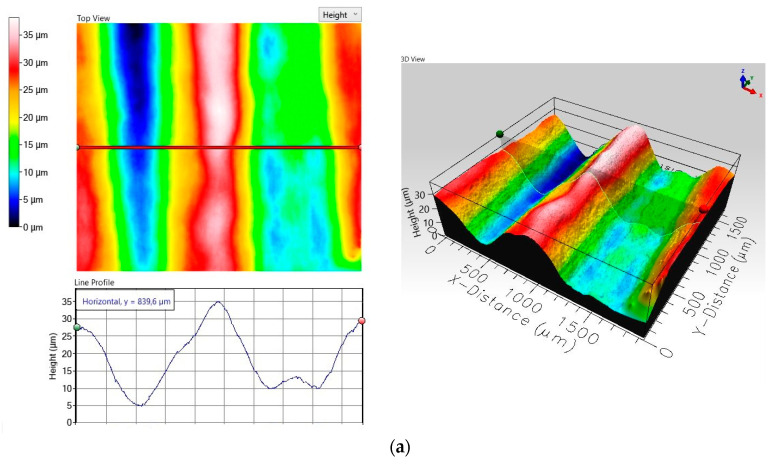

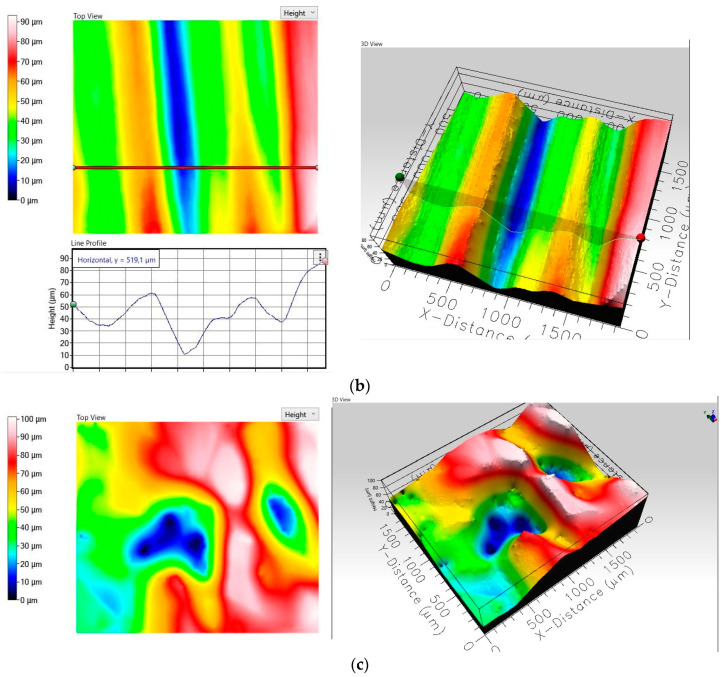
View of the surface of the representative sample after the metal-mineral abrasive wear resistance test observed under a confocal microscope: (**a**) HCCr wear plate; (**b**) CDP^®^ 4666 wear plate; (**c**) PHWP wear plate.

**Figure 11:**
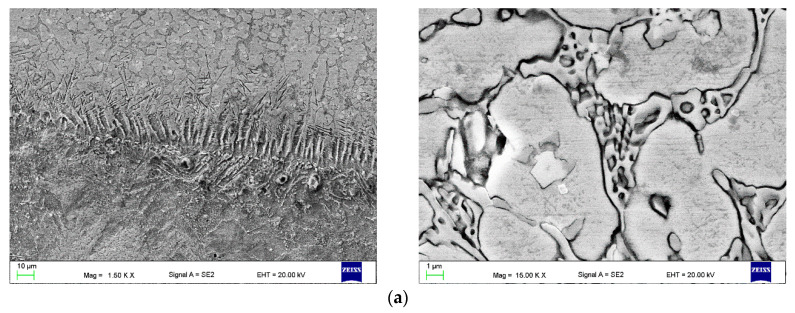

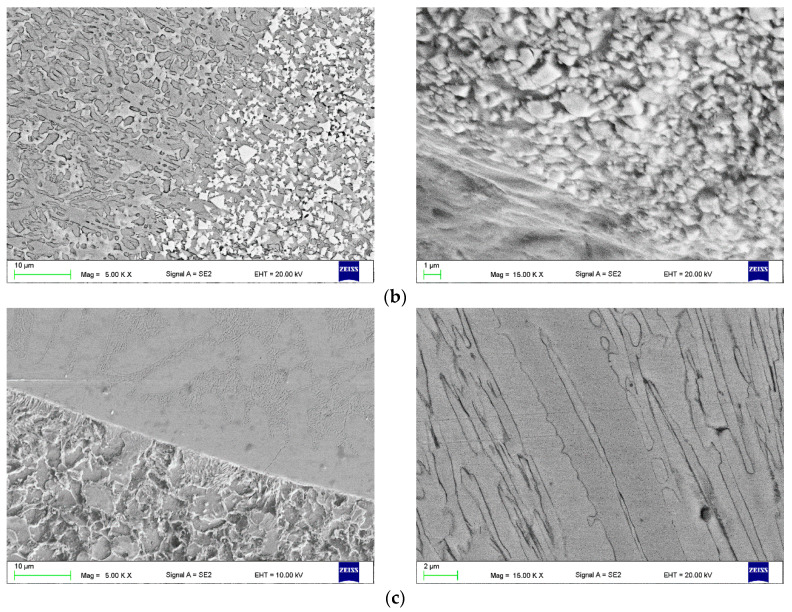
View of the surface of the representative sample after the metal-mineral abrasive wear resistance test observed under a scanning electron microscope: (**a**) HCCr wear plate; (**b**) CDP^®^ 4666 wear plate; (**c**) PHWP wear plate.

**Figure 12:**
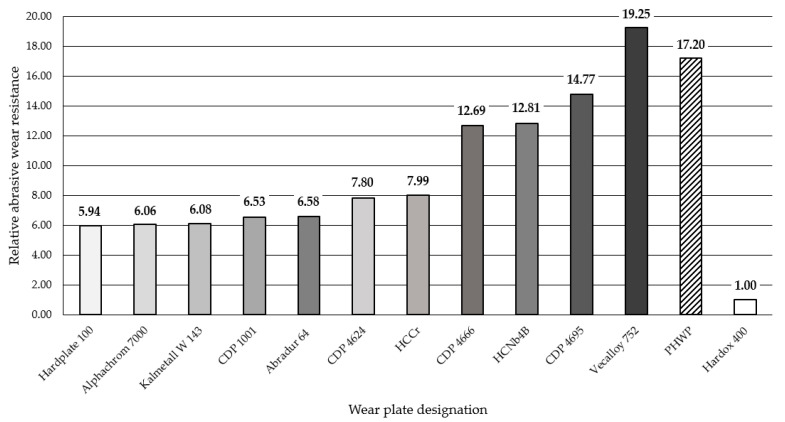
Relative metal-mineral abrasive wear resistance (ASTM 65-00, procedure A) of wear plates hardfaced with alloys from groups T Fe15 and T Fe16 in relation to Hardox 400 abrasive wear resistance.

The results obtained in the metal-mineral wear resistance test of selected wear plates hardfaced with alloys from groups T Fe15 and T Fe16 (Table 3) were compared to the wear resistance of a Hardox 400 steel plate, resulting in a relative abrasive wear resistance number. The surface view of the samples after the metal-mineral abrasive wear resistance test performed according to ASTM G65-00: Procedure A is presented in Figure 9. Selected surface areas of the representative samples after the metal-mineral abrasive wear resistance test observed under a confocal microscope and scanning microscope are presented in Figure 10 and Figure 11. Among the composite hardfaced wear-resistant plates examined, two produced by the automated FCAW-GS method, with trade names CastoDur Diamond Plate^®^ 4695 and Vecalloy 752 Plate^®^, deserve special attention. The corresponding relative abrasive wear resistance was 15 and 19 times higher than the Hardox 400 reference material. The average results of mass loss in the ASTM 65 test of the CastoDur Diamond Plate^®^ 4695 and Vecalloy 752 Plate^®^ plates were presented by Górka et al. [45] and Gucwa et al. [46]. Under the conditions of the experiment performed, the lowest relative metal-mineral abrasive wear resistance was achieved by the hardfaced composite wear-resistant plate under the trade name Hardplate™ 100S (Figure 12). The average value of the metal-mineral wear resistance of the PHWP plate was higher than the result obtained for the CastoDur Diamond Plate^®^ 4695 plate made by NanoAlloy using the FCAW-SS automated hardfacing process.

### 3.4. Metallographic Test Results and Results of the X-ray Diffraction Analysis

**Figure 13:**
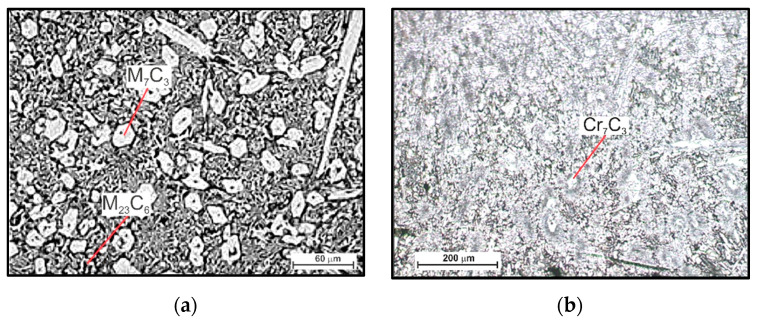

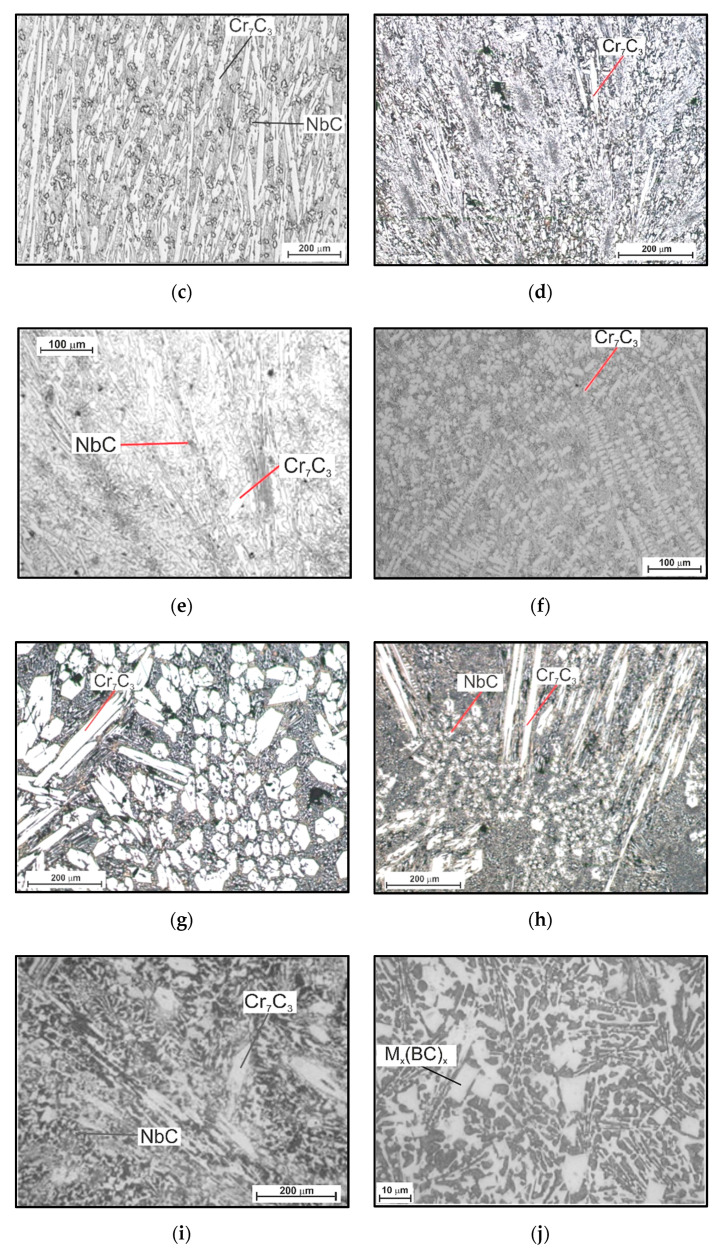

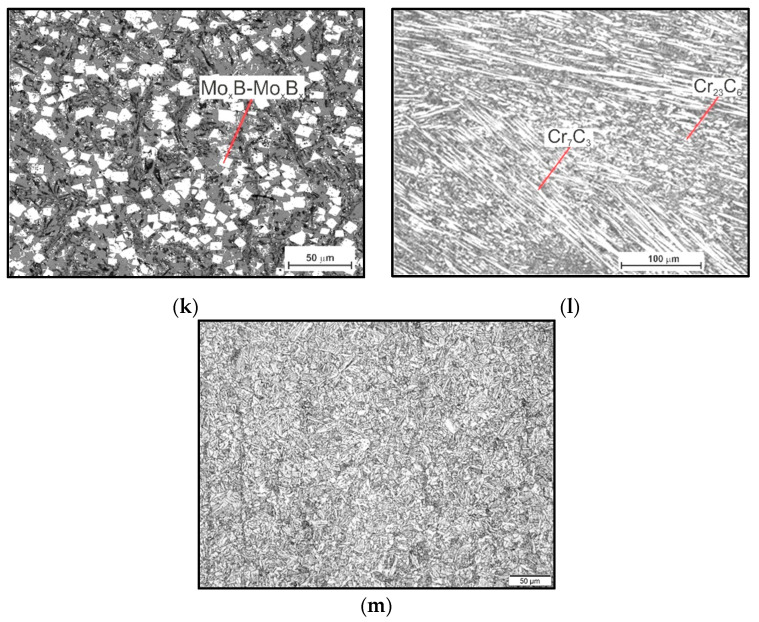
Microstructure of wear-resistant material of wear plates hardfaced with alloys from groups T Fe15, T Fe16, and the reference material: (**a**) Hardplate™ 100S wear plate; (**b**) Alphachrom 7000 wear plate; (**c**) Kalmetall W 143 wear plate; (**d**) CDP^®^ 1001 wear plate; (**e**) Abradur 64 wear plate; (**f**) CDP^®^ 4624 wear plate; (**g**) HCCr wear plate; (**h**) CDP^®^ 4666 wear plate; (**i**) HCNb4B wear plate; (**j**) CDP^®^ 4695 wear plate; (**k**) Vecalloy 752 Plate wear plate; (**l**) wear plate PHWP; (**m**) Hardox 400 abrasion-resistant steel.

**Figure 14:**
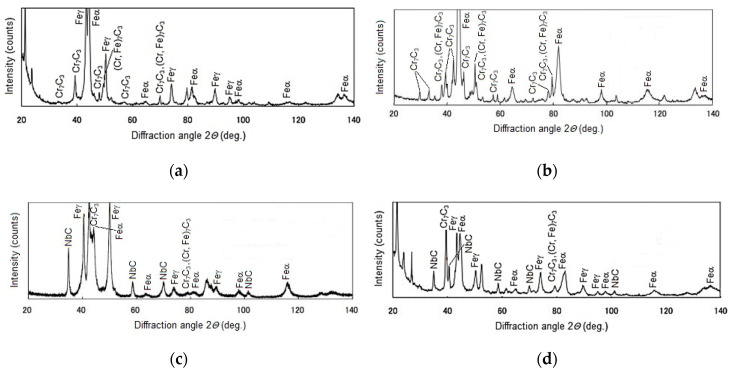

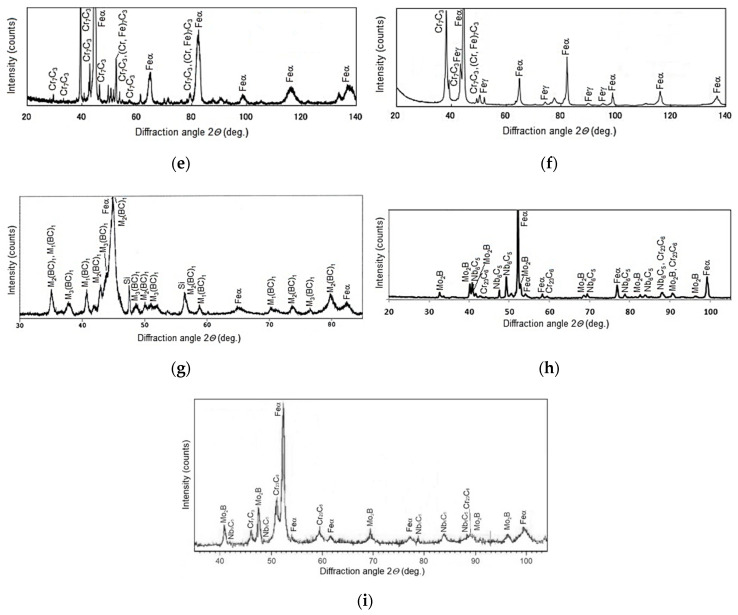
Selected diffractograms of the wear-resistant material of wear plates hardfaced with alloys from groups T Fe15 and T Fe16: (**a**) Hardplate™ 100S wear plate; (**b**) Alphachrom 7000; (**c**) Kalmetall W 145 wear plate; (**d**) Abradur 64; (**e**) CDP^®^ 4624 wear plate; (**f**) HCCr wear plate; (**g**) CDP^®^ 4695 wear plate; (**h**) Vecalloy 752 Plate^®^ wear plate; (**i**) PHWP wear plate.

**Figure 15:**
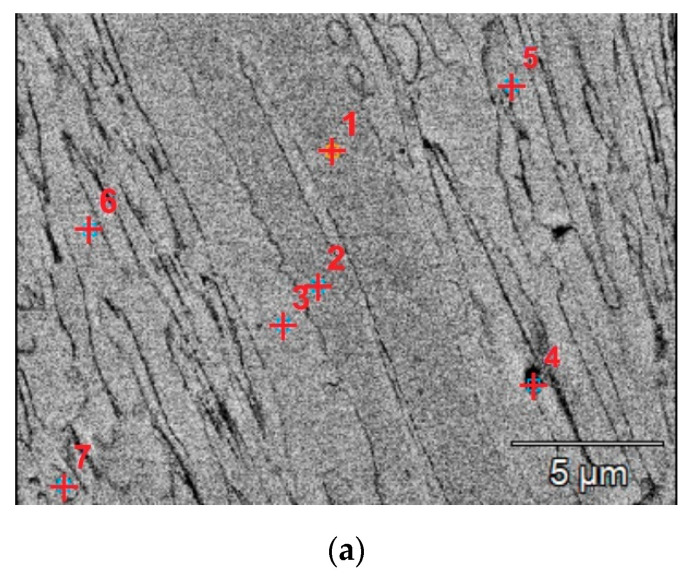

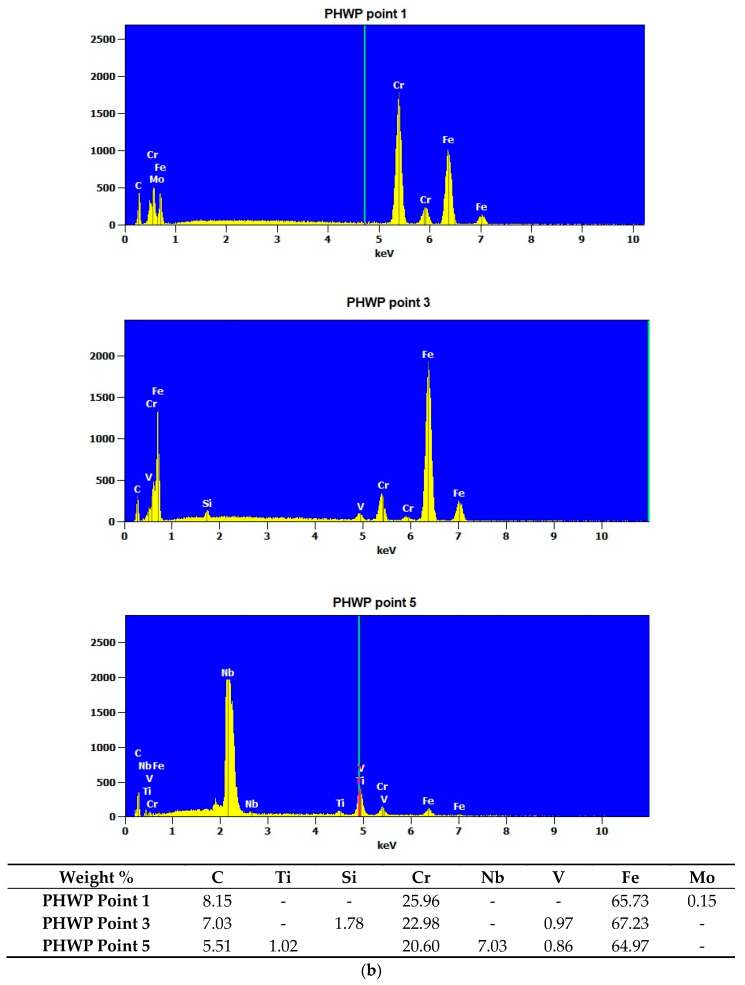
Sample BSE (Back Scattered Electrons) image of the surface layer microstructure with the results of EDS (Energy Dispersive Spectroscopy) point microanalysis, mag., 15,000×, high tension, 20 kV (PHWP sample): (**a**) view of the carbide structure; (**b**) point chemical analysis of the study area (measurement points: 1, 3, 5).

The microscopic metallographic examinations enable the determination of the microstructures of the plated layer of wear-resistant plates hardfaced with alloys from groups T Fe15 and T Fe16 and the Hardox 400 steel reference material (Figure 13). Moreover, X-ray diffraction analysis allowed determining the phase composition. The selected diffractograms are presented in Figure 14. The results of X-ray diffraction analysis were confirmed by means of energy-dispersive spectroscopy (EDS) (Figure 15).

The metallographic analysis of the wear-resistant plates hardfaced with an abrasion-resistant layer of group T Fe 15 alloys revealed the existence of the martensitic Fe-Cr-C ternary system phase in all of the samples and an austenitic Fe-Cr-C ternary system phase in some of the samples. These two phases formed a metal matrix. For the hardfaced layers with a high chromium cast alloy composition, it is presumed that a primary austenitic phase was partially transformed into a martensitic phase during the hardfacing thermal cycle. The degree of transformation varied among the examined wear-resistant plates. Several metal carbides can be observed in the X-ray diffractogram patterns. The main identified metal carbide in all the layers produced by hardfacing with alloys from group T Fe15 is the primary Cr_7_C_3_ carbide, and Cr_23_C_6_ eutectic carbides can sometimes be observed. Moreover, in the abrasion-resistant layers of Kalmetall W 143, Abradur 64, CastoDur Diamond Plate^®^ 4666, and HCNb4B plates, indices of NbC phases were found. The microstructures of abrasion-resistant layers hardfaced with alloys from group T Fe16 demonstrated a more complex composition characterized by the presence of borocarbides and molybdenum borides.

The hardfaced layer of the Hardplate™ 100S wear-resistant plate (Figure 13a) was characterized by a chromium cast iron structure with a fine Cr_7_C_3_ carbide precipitation of approximately 20 µm and a microhardness in the range of 950–1450 HV, uniformly distributed in the austenitic matrix (Figure 14a). The weld metal dilution was around 22%.

A similar microstructure was observed in the Alphachrom 7000 wear-resistant plate (Figure 13b). The hardfaced layer was characterized by an austenitic structure with a precipitation of primary Cr_7_C_3_ chromium carbides oriented perpendicularly to the surface (Figure 14b). The weld metal dilution was the highest and exceeded 25%.

In contrast, the hardfaced layer of the Kalmetall W 145 wear-resistant plate (Figure 13c) was identified as a chromium and carbon-rich iron alloy with a hard martensitic matrix structure, with 30 a vol % of primary Cr_7_C_3_ chromium carbides, a microhardness around 2200 HV, and niobium carbides NbC with a microhardness around 2400 HV (Figure 14c). The carbides were oriented parallel to the working surface, which increased abrasive wear resistance. The calculated weld metal dilution was under 24%.

The microstructure of the hardfaced layer of the CastoDur Diamond Plate^®^ 1001 wear-resistant plate (Figure 13d) was composed of primary Cr_7_C_3_ chromium carbides with a microhardness in the range of 1500–2200 HV and M_2_B metal borides with a microhardness of 1800 HV, regularly and densely situated in a plastic metal matrix. The base metal content was low, which promotes uniform and high abrasive wear resistance on the whole cross-section [26,47]. The hardfaced layer was distinguished by the lowest weld metal dilution rate of just over 19%. Abrasive wear of the surface layer of the wear plate resulted from microcutting. In the area of the abrasion, material discontinuities were present, caused by shearing unevenness of the surface with abrasive. The hardfaced layer of the Abradur 64 wear-resistant plate was characterized by a hypereutectic microstructure with chromium and niobium carbide precipitations (Figure 13e). The metallographic examination revealed the existence of a buffer layer with a high chromium, nickel, and boron content, presumed to act as a barrier for crack development. The MMA hardfaced wear-resistant layer of the Abradaur 64 plate, similar to the CastoDur Diamond Plate^®^ 1001 plate, was characterized by a uniform distribution of chromium and niobium carbides on the whole cross-section (Figure 14d). The weld metal dilution under 21%.

The hardfaced layer of the CastoDur Diamond Plate^®^ 4624 wear-resistant plate (Figure 13f) was characterized by a high chromium cast alloy microstructure of approximately 30 vol % of primary Cr_7_C_3_ chromium carbides (Figure 14e) 1800–2000 HV in hardness, uniformly distributed in the matrix. The carbide volume and distribution positively impacted the abrasive wear resistance of the plate. The weld metal dilution was slightly above 21%.

In contrast, the matrix of the hardfaced layer of the HCCr wear-resistant plate (Figure 13g) was austenitic in structure, with a high volume (over 50 vol %) of precipitations, mainly Cr_7_C_3_ chromium carbides (Figure 14f). Due to the high chromium and carbon content in the weld, a metal hardfaced layer with high abrasion resistance was achieved. The weld metal dilution was under 23%.

The above-characterized wear-resistant layers were subjected to intensive abrasive wear. The volumetric material loss was in the range of 23–32 mm^3^. The wear mechanism was deeply grooved, with plastic deformation of the contact area and raising of both groove edges (Figure 9a,b) or microcutting (Figure 9c–g). The wear was accompanied by the formation of surface defects, e.g., scuff marks or microcraters.

The hardfaced layer of the CastoDur Diamond Plate^®^ 4666 wear-resistant plate (Figure 13h) was characterized by a supereutectic ferrous alloy microstructure with a high chromium content. The microstructure was composed of a high fraction (over 50 vol %) of hard (2000–2100 HV) Cr_7_C_3_ chromium carbides, niobium carbides NbC, and chromium borides Cr_2_B, as well as other metal compounds distributed in a hard–austenitic matrix. The calculated weld metal dilution was under 21%.

The hardfaced layer of the HCNb4B plate (Figure 13i) was characterized by an alloyed austenitic microstructure with optimally oriented Cr_7_C_3_ chromium carbide precipitations and hard niobium carbides NbC and a hardness of approximately 1800 HV. The weld metal dilution was over 21%.

The structure of overlays produced on the CastoDur Diamond Plate^®^ 4695 wear plate (single weld layer on a carbon steel substrate) is presented with eight micron-scale images in Figure 13j. The high volume fraction of the ultrahard complex borocarbides (M_23_(BC)_6_), metal carbides (MC), and metal borides (M_2_B) is finely distributed within a mesomorphous α–Fe alloy matrix (Figure 14g). The abrasion-resistant layer possessed very high abrasive wear resistance due to the in situ formation of a high fraction of the complex borocarbide phase (−70 vol %). The weld metal dilution was over 22%.

The FCAW-GS hardfaced layer of the Vecalloy 752 Plate^®^ wear-resistant plate was unique in the microstructure of the fine martensitic ferrous alloy (Figure 13k). The microstructure was similar to composite layers reinforced with tungsten carbide (WC). The microstructure was composed of cubic molybdenum borides (33–50 vol %), with a hardness on the level of tungsten carbide, embedded in a hard martensitic matrix with a high fraction of primary and eutectic Cr_7_C_3_ and Cr_23_C_6_ chromium carbides, niobium carbides, and metal borides (Figure 14h). The size of the complex molybdenum borides was around 10 µm, with spacing lower than 50 µm. The weld metal dilution was around 23%. The slightly lower abrasion wear resistance of the wear plate CDP 4695 compared to the Vecalloy 752 wear plate can be explained by the lesser volume fraction of stable borocarbides, carbides, and borides in the deposit.

The hardfaced layer of the PHWP wear-resistant plate (Figure 13l) was characterized by a highly alloyed chromium carbide microstructure with a high concentration of complex carbides. The microstructure was composed of a high eutectic fraction of hard (1000–1600 HV) Cr_23_C_6_ chromium carbides; niobium carbides NbC, with a hardness of approximately 1800 HV and molybdenum borides Mo_2_B; and other metal compounds distributed in a plastic austenitic–martensitic matrix (Figure 14i).

Advantageous properties under the conditions of abrasion by mineral grains and erosion were also revealed by padding welds made of iron-based materials in the Fe-Cr-C-Mo-B alloy. The addition of boron increases the flowability and wettability of the metal and lowers the melting point. The abrasive wear resistance of borides was much higher than that of carbides. According to Hejowski [36], an increase in the boron content with constant chromium content increases the volume fraction and hardness of the primary phases. The calculated weld metal dilution was the smallest and amounted to 17%.

The wear mechanism of the wear-resistant layers presented in Figure 9h–l consisted only of the microcutting of the contact surface by abrasive grains. A volumetric loss of 10 to 15 mm^3^ was measured. As a result of the wear process, strengthening phases embedded in the ductile matrix were revealed.

### 3.5. Linear Regression Model

In the majority of cases, the increase in hardness coincides with the increase in wear resistance, especially in hardfaced wear plates. Research conducted by Marulanda-Arévalo et al. [1] and Ban et al. [4] demonstrated a strong linear relationship between the hardness and the wear resistance of the hardfaced wear plates. ASTM G 64 tests were performed on samples that had undergone a grinding treatment. The surface condition of the wear plate was taken into account for the purpose of determining wear resistance.

**Figure 16:**
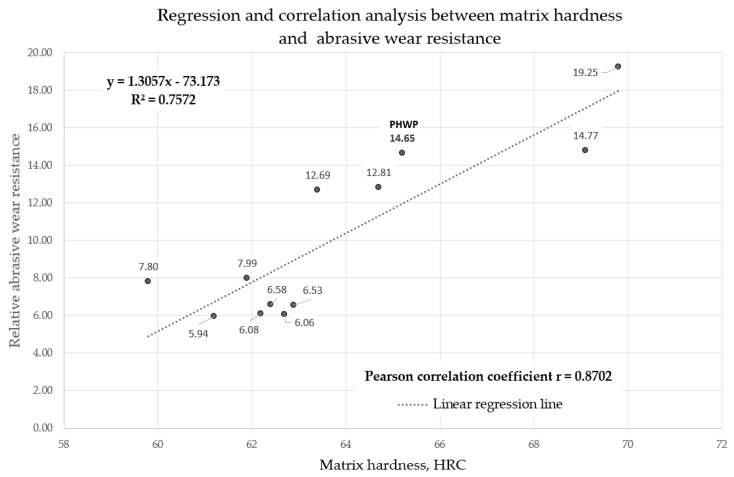
Regression and correlation analysis between matrix hardness and abrasive wear resistance.

The obtained abrasive wear resistance test results of arc-hardfaced alloys from groups T Fe15 and T Fe16 were used to obtain a mathematical linear regression model describing the dependence between metal-mineral wear resistance and the hardness of hardfaced layers. A linear predictor function with an equation in the form y = a + bx, where x is the explanatory variable (surface hardness) and y is the dependent variable (abrasive wear resistance), was used (Figure 16).

Pearson’s r correlation coefficient was calculated for the proposed model and was equal to 0.8702, which indicates the high accuracy of the description of the linear model between the hardness of the layer and its resistance to abrasive wear. The calculated coefficient of determination R² = 0.7572 proves the accuracy of the model. The proposed mathematical linear regression model describes the variability of the dependent variable (abrasive wear resistance) and the independent variable (surface layer hardness) by nearly 80%.

## 4. Conclusions

The aim of this research was to compare abrasive wear resistance and determine the linear correlation between surface hardness and the metal-mineral wear resistance of 11 commercially produced and industrially applied wear-resistant composite plates and hardfaced layers produced by a patented covered tubular electrode with a special chemical composition of the metallic core. The comparative analysis allowed for the formulation of the following conclusions:

1. The layers of commercially produced, arc-hardfaced, additional modern materials from alloy group T Fe16 composite wear-resistant plates possess higher hardness and abrasive wear resistance compared to conventional chromium carbide overlay (CCO) wear plates with layers hardfaced with alloys from the T Fe15 group. The higher wear resistance of the CastoDur Diamond Plate^®^ 4695, Vecalloy 752 Plate^®^, and PHWP plates results from the higher volume fraction and dispersion of more uniform, finer, and harder precipitations in the matrix. For chromium carbide overlay (CCO) wear plates with layers hardfaced with alloys from the T Fe15 group, the more malleable metal matrix was subjected to more intensive wear from abrasive interaction. This revealed brittle carbides, causing cracking and ripping of the carbides, promoting further degradation of the matrix. The type, size, and dispersion of strengthening phases further impact abrasive wear resistance.
2. The metallographic examination and X-ray diffraction analysis indicated that layers hardfaced with alloys from the T Fe15 group have microstructures composed mostly of primary M_7_C_3_ carbides uniformly distributed in the Fe-Cr-C-type matrix, with occasional harder NbC-type carbides. The microstructure of the layers hardfaced with alloys from the T Fe16 group was more complex and consisted of ultrahard complex borocarbides M_23_(BC)_6_, MC metal carbides, M_2_B metal borides, or nearly cubic complex Mo_2_B molybdenum borides, Cr_7_C_3_ primary chromium carbides, Cr_23_C_6_ eutectic chromium carbides, and Nb_6_C_5_ niobium carbides densely distributed in a martensitic matrix.
3. The metal-mineral abrasion resistance of layers made by the covered tubular electrode with an innovative chemical composition of metal core is equal to the plate made in the FCAW-SS process with the cored electrode wire EnDOtec DO*395N, which, according to the manufacturer, allows obtaining a nanostructured weld metal.
4. The obtained results suggest the high linear relationship between an increase in surface hardness increase and an increase in the metal-mineral abrasive resistance of the wear-resistant plates’ hardfaced layers. The developed linear regression model will be applied in real-time wear prediction systems for wear plates and liners. It is worth noting that no factors, such as the type and chemical composition of the additional material, distribution, and geometric properties of the strengthening phase, were considered.

In the future, the results of laser and plasma layers hardfaced with alloys from the Ni20 group and metal-mineral abrasive wear resistance will be published by the author.

## Figures and Tables

**Table 1:** Hardfaced composite wear-resistant plates with an arc hardfaced layer by T Fe15 and T Fe16 alloys.

Name of the Hardfacing Wear Plate	Group of Alloy in Accordance with EN 14700 [30] Element Contents Hardfacing Technology Typical Applications and Industry
Hardplate™ 100S	T Fe15 C (5.0%), Cr (27.0%), Mn (1.5%), Si (1.5%), Fe (Balance) Self-shielded flux-cored arc welding (FCAW-SS) Applications: mill shell liners, dump truck bin liners, skid protection on loader buckets, and grinding components Industry: cement, mining, power, ceramic, transport, construction
Alphachrom 7000	T Fe15 C (5.4%), Cr (27.0%), Mn (0.2%), Si (1.3%), Fe (Balance) Self-shielded flux-cored arc welding (FCAW-SS) Applications: chute linings, sieve plates, drum linings, augers, crusher components, mixer linings, and crusher Industry: cement, ceramic, quarries, construction, transport, mining, and power
Kalmetall W 143	T Fe15 C (5.0%), Cr (27.0%), Nb (5.0%), Fe (Balance) Self-shielded flux-cored arc welding (FCAW-SS) Applications: screw conveyors, ventilator housings, cyclones, separators, mixer linings, piping components, screens, troughs, and transport channels Industry: cement, ceramic, quarries, construction, transport, mining, and power
CastoDur Diamond Plate^®^ 1001	T Fe15 C (xx%), Cr (xx%), B (xx%), Fe (Balance) Self-shielded flux-cored arc welding (FCAW-SS) Applications: crusher hammer-breaker bars, secondary crushers, chutes, earthmoving equipment, bucket parts, conveyor chain-crushers, conveyor screw-mill augers, and road repair equipment Industry: cement, disposal, quarries, construction, mining, paper, and power
Abradur 64 ^1^	T Fe15 C (7.0%), Cr (24.0%), Nb (7.0%), Fe (Balance) Manual metal arc welding (MMAW) Applications: cane knives, dozer blades, bucket teeth for excavators, worm wheels, and other mining equipment Industry: cement, quarries, construction, mining, transport, and agriculture
CastoDur Diamond Plate^®^ 4624	T Fe15 C (xx%), Cr (xx%), Mn (xx%), Si (xx%), Fe (Balance) ^3^ Self-shielded flux-cored arc welding (FCAW-SS) Applications: fan blades and housings, cyclones, pipe, and pipe elbows Industry: cement, steel, utilities, oil and sand mining, waste and recycling, pulp and paper
HCCr	T Fe15 C (4.8%), Cr (32.0%), Mn (1.5%), Si (0.8%), Fe (Balance) Self-shielded flux-cored arc welding (FCAW-SS) Applications: mixer blades and components, pipes, scrapers, and mills Industry: cement, ceramic, power, transport, construction, and agriculture
CastoDur Diamond Plate^®^ 4666	T Fe15 C (4.8%), Cr (32.0%), Mn (1.5%), Si (0.8%), B (0.3%), Fe (Balance) Self-shielded flux-cored arc welding (FCAW-SS) Applications: mill linings, pipelines, coal bunker, chain conveyor, bucket, crusher, tans, cyclones, and blast furnace gas systems Industry: power, cement, mining, steel, paper and pulp
HCNb4B	T Fe15 C (4.5%), Cr (23.0%), Nb (4.5), Mn (1.5%), Si (0.8%), B (0.4%), Fe (Balance) Self-shielded flux-cored arc welding (FCAW-SS) Applications: mixer blades, mills, and chutes Industry: cement, ceramic, power, transport, and construction
CastoDur Diamond Plate^®^ 4695	T Fe16 C (5.0%), Cr (20.0%), Mo (10.0%), Nb (10.0%), W (10.0%), Mn (5.0%), B (5.0%), Si (2.0%), Fe (Balance) Flux-cored arc welding gas-shielded (FCAW-GS) Gas mixture type Ar-CO_2_ Applications: screws, rotor and housing, feeding tube, knives and wear pieces, hammers, fan blades, and separators Industry: waste and recycling, cement, ceramic, power, quarries and mining
Vecalloy 752 Plate^®^	T Fe16 C (3.5%), Cr (11.5%), Mo (1.2%), Nb (5.0%), B (4.8%), Fe (Balance) Flux-cored arc welding gas-shielded (FCAW-GS) Argon gas Applications: shaker screens, grader blades and other ground-engaging tools, chute blocks, inner pipe walls, mill liners, slurry pipes, shovel wear packages, cutter rings, primary and secondary crusher teeth, grouser risers, and other mining applications Industry: cement, agriculture, quarries, mining, ceramic, and power
PHWPPatented hardfacing wear plate ^2^	T Fe16 C (5.0%), Cr (23.0%), Mo (xx%), Nb (xx%), Si (xx%), B (xx%), W (xx%), V (xx%), Fe (Balance) ^3^ Manual metal arc welding (MMA) with patented cover tubular electrode Potential applications: screeners, blast furnace hoppers, extractor fans Industry: iron and steel, power, quarries, and cement

Remarks: ^1^ Surface layer manufactured in a three-pass process; ^2^ surface layer manufactured in a one-pass process; ^3^ chemistry composition proprietary to its own copyright patent [31].

**Table 2:** Characteristics of the abrasion-resistant steel.

Name of the Abrasion-Resistant Steel	Steel in Accordance with EN 10029 [32] Element Contents Metal Hardness
Hardox 400 Steel	C (0.32%), Si (0.7%), Mn (1.6%), P (0.025%), S (0.01%), Cr (1.4%), Ni (1.5%), Mo (0.6%), B (0.004%), Fe (Balance) 38–44 HRC (372–435 HV)

**Table 3:** Abrasive wear resistance test results of wear plates hardfaced with alloys from groups T Fe15 and T Fe16 in relation to the Hardox 400 abrasive wear resistance.

Specimen Designation	Spec. Number	Mass Before Test, g	Mass After Test, g	Mass Loss ^1^, g	Average Mass Loss, g	Clad Layer Density, g/cm^3^	Average Volume Loss, mm^3^	Relative Abrasive Wear Resistance ^2^	Diluted Weld Metal, %
Hardplate P100S	p01 p02	130.6009 129.6213	130.3432 129.4118	0.2577 0.2095	0.2343	7.3229	31.9955	5.94	22.1
Alphachrom 7000	p01 p02	157.5045 157.0426	157.2484 156.8331	0.2561 0.2095	0.2328	7.4324	31.3223	6.06	25.4
KalmetallW 143	p01 p02	91.8884 87.1791	91.5677 86.8916	0.2575 0.2107	0.2341	7.4943	31.2371	6.08	23.6
CDP 1001	p01 p02	173.3469 178.7772	173.1099 178.5833	0.2371 0.1940	0.2155	7.4139	29.0670	6.53	19.1
Abradur 64 ^3^	p01 p02	136.2893 139.6675	136.0602 139.4800	0.22913 0.18747	0.2083	7.2144	28.8728	6.58	20.7
CDP 4624	p01 p02	134.2440 134.0617	134.0815 133.8630	0.1625 0.1987	0.1806	7.4191	24.3426	7.80	21.0
HCCr	p01 p02	172.0538 160.5386	171.8583 160.3787	0.1955 0.1599	0.1777	7.4756	23.7707	7.99	22.8
CDP 4666	p01 p02	161.9005 164.5962	161.7996 164.4729	0.1009 0.1233	0.1121	7.4894	14.9678	12.69	20.7
HCNb4B	p01 p02	174.3838 176.8729	174.2856 176.7529	0.0982 0.1200	0.1091	7.3559	14.8316	12.81	21.3
CDP 4695	p01 p02	155.4632 155.8611	155.3738 155.7519	0.0894 0.1092	0.0993	7.7208	12.8614	14.77	22.5
Vecalloy 752	p01 p02	167.8436 168.2761	167.7602 168.2079	0.0834 0.0682	0.0758	7.6816	9.8677	19.25	22.7
PHWP ^4^	p01 p02	161.8431 163.5409	161.7440 163.4401	0.0991 0.1008	0.0999	7.7112	12.9617	14.65	17.2
Hardox 400	p01 p02	116.2260 116.7526	114.7526 115.2773	1.4734 1.4753	1.4744	7.7620	189.9510	1.00	-

Remarks: ^1^ Mass loss in 30 min; ^2^ relative abrasive wear resistance to Hardox 400 steel; ^3^ surface layer manufactured in a three-pass process; ^4^ surface layer manufactured in a one-pass process.
